# Pulmonary Hydatid Cyst in Children and Adults: Diagnosis and Management

**DOI:** 10.5152/eurasianjmed.2022.22289

**Published:** 2022-12-01

**Authors:** Yener Aydin, Ali Bilal Ulas, Ayman Gaffar Ahmed, Atilla Eroglu

**Affiliations:** 1 Department of Thoracic Surgery, Ataturk University, Medical Faculty, Erzurum, Turkey; 2 Department of Thoracic Surgery, King Abdullah Medical City, Makkah, Saudi Arabia

**Keywords:** Hydatid cyst, lung, children, adults, radiology, surgery

## Abstract

Hydatid cyst disease induced by *Echinococcus granulosus* is a parasitic disease known since ancient times. Today, it continues to be seen in many countries and creates serious problems. The lung is the second most frequently affected organ by hydatid cysts after the liver. Lung involvement is more prevalent in children than adults, and the growth of the cyst is faster in children. Hydatid cysts are mostly seen in the right lower lobe of the lung. Common symptoms are chest pain, cough, and shortness of breath, with the most diagnostic symptom being the expectoration of cyst fluid or membranes. In endemic areas, the diagnosis of hydatid cysts can usually be made easily by clinical findings, serology tests, and radiological findings. When the hydatid cyst ruptures and becomes complicated, it is clinically and radiologically confused with many diseases, especially lung cancer. Surgery is accepted as primary treatment of lung hydatid cysts all over the world. The surgical approach is related to several factors such as the size of the cyst, whether it is intact or complicated, unilateral or bilateral, solitary or multiple, and the presence of destruction of the lung parenchyma. Although it is stated by some surgeons that capitonnage is not required, the most frequently applied surgical technique is cystotomy and capitonnage. Pulmonary resection should be avoided as much as possible, particularly in children. Albendazole or mebendazole treatment in pulmonary hydatid cyst is generally used after surgery and to prevent recurrences.

**Main Points**

- Hydatid cyst (HC) disease is an important health problem today.
- Lung is the second most frequently involved organ, and bilateral involvement is detected in approximately 15% of cases.
- When the HC ruptures and the cyst fluid and membrane pass into the bronchus, it can cause serious complications such as pneumonia, asphyxia, and anaphylaxis.
- The primary treatment of pulmonary HC is surgery, and cystostomy and capitonnage are the most common methods.
- In HCs of the liver dome and right lung, surgery can be performed on both the lung and the liver in a single session with a transthoracic approach.

## Introduction

Hydatid cyst (HC) is a disease known since the time of Hippocrates. It is a zoonotic disease that is often seen in regions where animal husbandry and agriculture are common and preventive measures are not taken. The causative agent of this disease is a parasite belonging to the cestode class *Echinococcus granulosus*.^1^

In recent years, the incidence of HCs has increased.^2^ The most frequently affected organ is the liver with a rate of 60%-80%, and the second most common is the lung with a rate of 20%-30%.^1,2^ The third is the spleen followed by the musculoskeletal system, kidneys, and the intracranial system.^2^ Although it is very rare, HC may be seen in any part of the body.

## Lifecycle and Transmission to Humans

The lifecycle of echinococci consists of a definitive host, generally dogs, and an intermediate host, like goats, sheep, camels, deer, pigs, cattle, and horses. Humans are fortuitous hosts and do not participate in the transmission loop. *E. granulosus* adult tapeworm is commonly seen in canines.^3,4^

Adult tapeworms live in the small intestines of definitive hosts. The exact host could be infected with thousands of parasites. *E. granulosus* parasites are usually 2-7 mm long and comprise a sucking and hooked scolex and at least 3 proglottid segments.^5^ The tapeworm consists of proglottid segments that have both female and male genitalia and can produce 30-40 µm-sized eggs containing oncosphere. Each adult parasite can produce thousands of eggs daily. These are released with the stool of the final host into the environment where sensitive intermediate hosts are infected. The eggs are very hard and can remain contagious for a year in a humid environment with low temperature.^5,6^

After egg ingestion by the intermediate host, oncospheres hatch and penetrate to the mucous membrane of the intestine. Then, it enters the lymphatic system and/or blood and goes to the liver or other organs. A few days later, a fluid-filled cyst starts to grow and then multiple layers develop into metacestodes. When definitive hosts ingest intermediate host’s organs comprising HCs composed of protoscolices, the protoscolices protrude, attach to the intestinal mucosa, and advance into adult parasites. This development takes place over 4-7 weeks, which completes its life cycle. Contamination often arises in environments where dogs eat the internal organs of slaughtered animals. Then they release infective eggs in their stool, which can be passed to humans and other animals via the fecal–oral route. This can occur through contamination of water and cultivated vegetables or contact between infected pet dogs and humans. Human-to-human transmission of echinococcosis does not occur as 2 mammalian species are required to complete its life cycle.^7,8^

## Epidemiology

The highest rates of cystic echinococcal endemic diseases tend to occur in areas where sheep are raised. Dogs in such areas often feed on offal and may not be depopulated for religious and other reasons. Cystic echinococcosis prevalence increases with age. Women are more often affected than men. This may be due to closer contact with dogs as a result of work such as feeding, herding, and milking farm animals. However, due to increased travel and migration rates, the disease can also be encountered sporadically.^9,10^

The *E. granulosus* sensu stricto G1 is the genotype that appears in most of all human cases. The hydatid cyst, which has a worldwide geographical distribution, is present in almost all continents. The parasite has the highest prevalence in parts of North Africa, Asia, South America, and Australia. Annual human incidence may exceed 50 per 100 000 in endemic areas, and prevalence rates of up to 5%-10% and can be seen in parts of China, East Africa, Argentina, and Peru.^11,12^

## Pathophysiology

The HC is usually filled with fluid. The germinative membrane is the inner layer that forms hydatid fluid and small secondary cysts that bud internally from this layer. Separation of the brood capsules and germinative layer leads to daughter cysts. Daughter vesicles are less common in the lung than in the liver. These may develop separately or within the original cyst. At least 10-12 months after infection, protoscolices are generated in brood capsules. The cysts containing protoscolex are fertile and may generate daughter cysts, while cysts without protoscolex are sterile. Apart from the germinative membrane, there is a variable thickness, cell-free, exocyst. A host granulomatous reaction occurs around this membrane, which results in a reaction of parenchyma and fibrous tissue known as pericyst.^13-15^

## Doubling Time

The growth rate of HCs is not fully understood. Calculating the doubling time of an HC is clinically very difficult. Bloomfield^16^ stated the doubling time of HCs as 16-20 weeks. Pedrosa et al^17^ stated that cysts in the human liver may grow by 1 cm in the first 6 months and 2-3 cm per year thereafter, depending on the host tissue resistance. It has been reported that HCs enlarge faster in children than adults, and a decrease in cyst growth rate is observed in pulmonary cysts with increasing host age.^18^

It is stated that HC grows more rapidly in the lung than in the liver because of the elastic structure of the lung. Although there are not enough investigations on this subject in the literature, clinical observations and experiences show that the doubling time of pulmonary HCs can be much faster than that of the liver.^19-21^

## Frequency of Pulmonary Involvement in Children and Adults

It has been reported that pulmonary HCs are more common in boys.^2,22^ Hydatid cysts are generally located in the liver. However, in many studies involving pediatric pulmonary HCs, it has been reported that the lung is the organ most frequently involved in children.^2^ This situation may be erroneous since relatively only lung cysts were examined and the general population was not calculated. In a large series of HCs in which 3090 cases were evaluated comprehensively in our hospital, the liver was the most frequently involved (66.87%) and the lung (38.59%) was the second most commonly involved organ in children. In adults, 84.94% of the liver and 17.38% of the lungs were involved. Also, in this study, male children were more frequently affected at 54.84% than adults at 40.1%.^2^

Hydatid cyst is typically referred to as a giant cyst when it is larger than 10 cm in diameter.^19^ Giant HCs are more common in young adults. This is indicated by the delayed onset of symptoms in this population due to more elasticity and cohesion of the lungs. ^19,23,24^

## Distribution of Pulmonary Localizations

**Figure 1. a-c.**
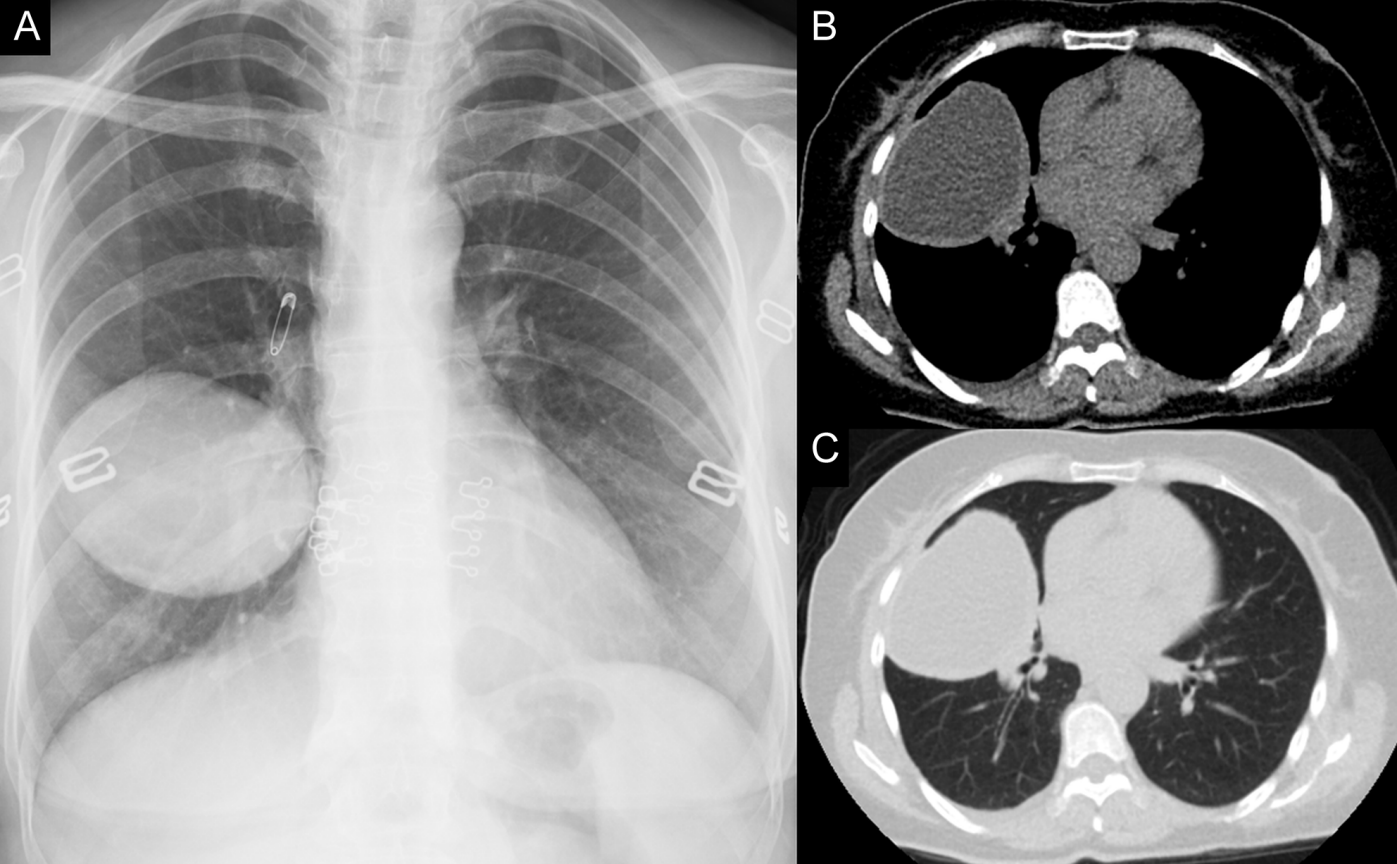
In a 60-year-old female, an intact hydatid cyst of approximately 9 cm in diameter originating from the lower lobe of the right lung is seen in posteroanterior chest x-ray (a), thorax computed tomography sections of the mediastinum (b) and parenchyma windows (c).

**Figure 2.**
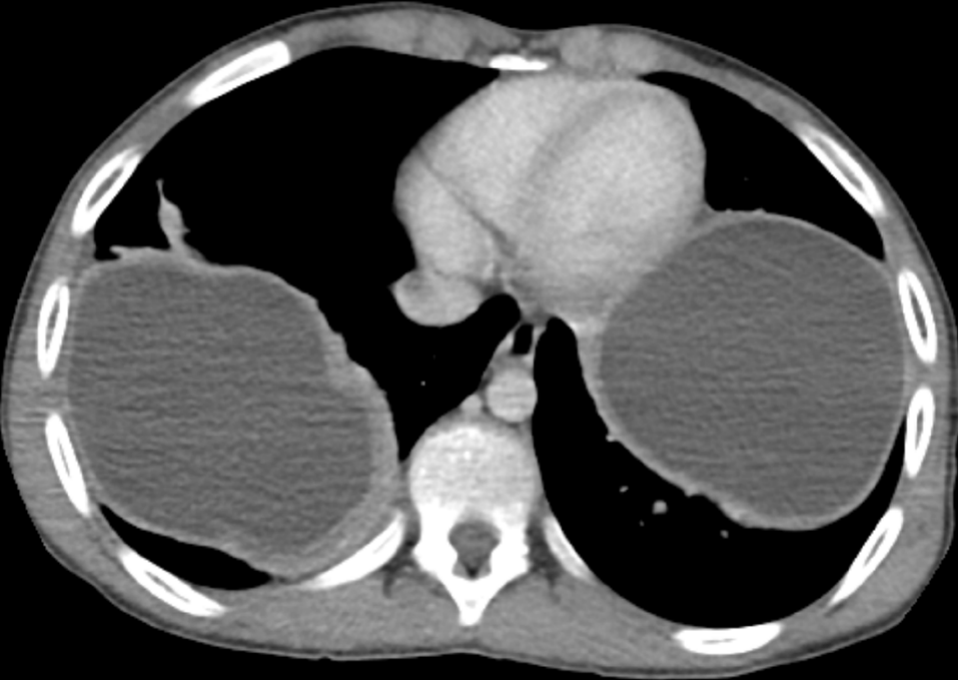
In a 13-year-old male, the image of giant hydatid cysts located in the lower lobe of the right lung and the lower lobe of the left lung is seen in the thorax computed tomography mediastinal window.

Single-organ involvement is seen in 85%-90% of patients with cystic echinococcus, and more than 70% have a solitary cyst^25^ (Figure 1). Since echinococcus usually enters the lungs through the liver, approximately 60% of pulmonary involvement involves the right lung and 50%-60% of cases involve the lower lobes.^26,27^ It has also been reported that HCs cause bilateral lung involvement in 4%-26%^28,29^ (Figure 2). In a large series study in our clinic, 608 (85.88%) of 708 cases with pulmonary involvement had single lung involvement and 100 (14.12%) had double lung involvement. On the other hand, when the pulmonary lobe distribution is considered, the involvement in the right lower lobe is seen in 329 (36.04%), 227 (24.86%) in the left lower lobe, 149 (16.32%) in the right upper lobe, 146 (15.99%) in the left upper lobe, and 62 (6.79%) in the right middle lobe.^2^

## Involvement of Other Organs with Lung

Liver cysts have also been reported in 20%-40% of patients with HCs.^30,31^ In addition, pleural space, mediastinum, and chest wall involvement may accompany lung in the thorax.^25^ In general, when all HC cases are evaluated, it is seen that the liver and lung are involved together in 8% of the cases, while approximately 38% of the cases with lung involvement are associated with the involvement of other organs, and in approximately 35% of them, the liver is also involved with the lung.^2^

## Clinical Symptoms and Signs

**Figure 3. a,b.**
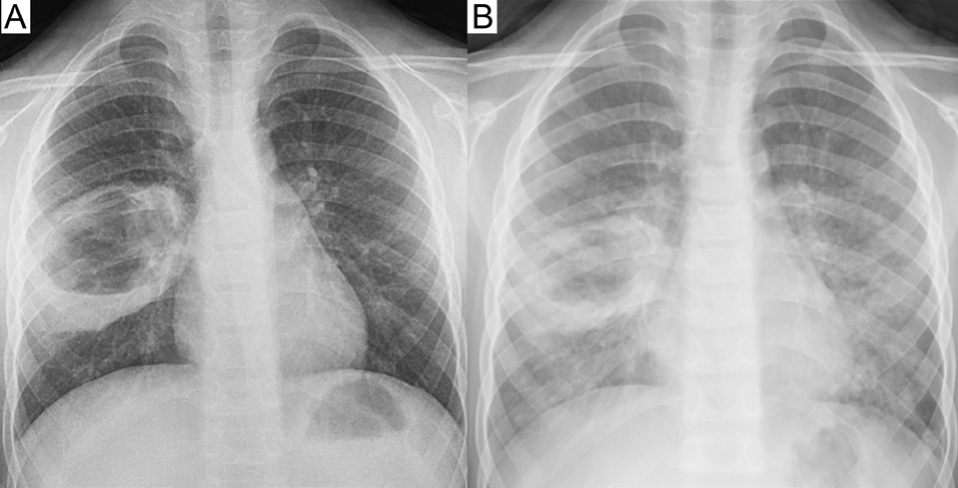
In a 7-year-old male who presented with cough and hydatoptysis, chest x-ray (a) taken at the time of admission and chest x-ray showing bilateral diffuse pneumonia 2 days after the perforation (b) are seen.

Pulmonary signs and symptoms are dependent on the size and location of the cyst. Intact and small cysts can remain asymptomatic, whereas, in giant HCs, symptoms involved mass compression to the neighboring structures, obstruction of lymphatic flow or blood, and findings related to cyst rupture can be seen. The most general symptoms in the literature for pulmonary HCs are cough (60%), chest pain (45%), dyspnea (25%), and hemoptysis (12%).^2,25,32,33^ The most diagnostic symptom is the expectoration of cyst membranes or fluid (hydatoptysis). Although hydatoptysis is pathognomonic, it is seen only in 7% of cases.^2^ This means that the cyst has perforated and opened into the bronchus. Perforation is the most general complication of lung HCs. Perforation rates are stated as 24.7%-61% in the literature.^2,19^ When the cyst perforates the pleura, it can cause hydropneumothorax, pneumothorax, secondary pleural hydatid disease, pleural thickening, empyema, and parenchymal destruction. Rupture of an HC to an adjacent bronchus can be manifested by serious coughing, large amounts of salty sputum, and sometimes expectoration of a piece of the laminar membrane (Figure 3). When the cyst ruptures, the patient may exhibit a serious hypersensitivity reaction represented by widespread rash, pulmonary congestion, high fever, and serious bronchospasm. Sometimes, intrabronchial rupture of the cyst presents with sudden and serious dyspnea, which can cause suffocation and death due to complete tracheal obstruction by the hydatid membrane fragments. The cyst content may be allergic to the patient and can lead to chemical pneumonia or anaphylactic shock.^34,35^

## Laboratory Findings

Even though non-specific mild eosinophilia, thrombocytopenia, leukopenia, and abnormalities in liver function tests are observed, they have no diagnostic value. Eosinophilia is seen in less than 15% of cases and mostly when antigenic cyst fluid is exposed to the environment.^36^

Immune responses in HC disease are quantitatively small. Therefore, routine serological tests are generally insufficient and can be used in the follow-up of treatment. The first immunological method used for the serological diagnosis of HC disease was the complement fixation test. Today, advanced techniques ranging from indirect hemagglutination (IHA) to radioimmunoassay and immunoblot are available. When the specificity and sensitivity of serological tests are compared, it is seen that enzyme-linked immunosorbent assay (ELISA) is the most sensitive and specific test. Hydatid antigen dot immunoassays are simple, inexpensive, and heat-resistant tests and are often used in field screening. Sensitivity is between 88% and 96%, and specificity is between 90% and 98%.^37-39^

There are 2 major *E. granulosus* antigens for serological testing. Antigen 5 is the main parasite antigen found in protoscolices, daughter vesicles, and the inner surface of the germinal layer. Antigen B is a very immunogenic polymeric lipoprotein, with higher specificity than using antigen 5. When these antigens are used with the ELISA, the sensitivity is 60%-90% and the specificity is almost 90%.^40,41^

Many factors affect false-negative and -positive results in serological tests. The first of these is the lack of standardization in laboratories, and the second is the different methods used in antigen isolation and purification. Combined or sequential use of tests increases the contribution of serology to diagnosis. In the first step, a highly sensitive ELISA or IHA test is often used, followed by a highly specific immunoblot or gel diffusion method to confirm the result. Also, choosing specific antibodies like immunoglobulin (Ig) G1 or IgG4 over total IgG increases specificity.^42,43^ Negative serological tests generally do not rule out an HC. Antibody response is more pronounced in liver HCs than in lung cysts. Taken as a whole, 85%-95% of liver cysts and 65% of lung cysts are accompanied by positive serology.^44^

## Radiological Findings

Pulmonary HC has very rich conventional chest radiography findings. Radiological findings are sometimes quite specific, and diagnosis may be possible in this way, especially in intact cysts. On posteroanterior chest radiographs, intact cysts appear as homogeneous, round or oval, well-circumscribed lesions surrounded by normal lung tissue. If the cyst has ruptured, specific findings may occur in the cystic lesion. However, consolidation sometimes occurs adjacent to the lesion, and the inflammatory reaction prevents a clear assessment of the lesion.^1,30,31^

The change in the size of intact cysts during inspiration and expiration is called the “Escurado–Nemerow sign.”^45^ Eggshell calcification is a radiological appearance that mostly develops as a result of the death of the cyst, and it is mostly seen in liver cysts and is very rare in the lung.^46^ “Moon sign” occurs when the pericyst layer is ruptured, as a result of air entering between the pericyst and endocyst layers and is a sign that the cyst will rupture.^47^ “Waterlily sign” occurs when the membrane floats in rock water as a result of rupture of the pericyst and endocyst.^45,48^ When both membranes are torn and some of the fluid is expectorated, the image forms as 2 air domes and a cyst membrane in between is called the “double-domed arch sign.”^49^ If the cyst fluid is completely drained, the germinal membrane shrinks in the cyst and gives a radiological appearance similar to a mushroom ball. The presence of small intracystic air bubbles between the endocyst and pericyst at the periphery of the cyst is called the “air bubble sign.”^50^

**Figure 4. a,b.**
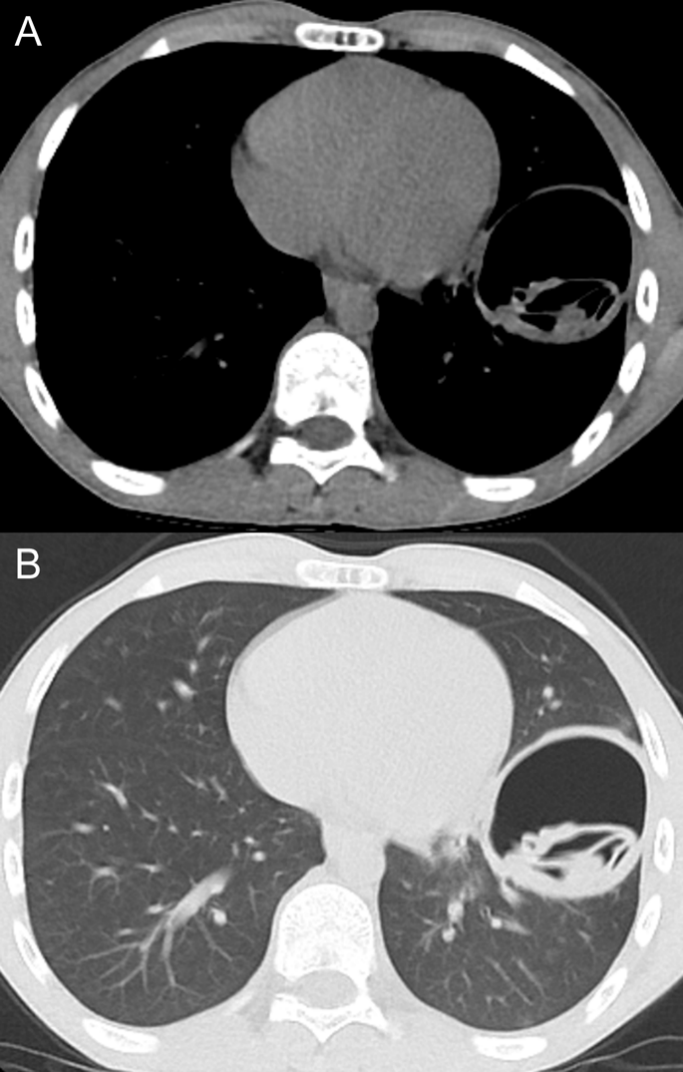
In a 14-year-old male, images of the air and laminar membrane in the cavity of the ruptured cyst in the left lower lobe are seen on the thorax computed tomography mediastinum (a) and parenchyma sections (b).

Computed tomography (CT) has an important role in the diagnosis of HCs in 2 aspects. The first is to determine the location and number of HCs correctly and to guide the surgeon before the surgical intervention. The second feature facilitates the differential diagnosis of complicated cysts that cannot be diagnosed by classical methods, with the wall thickness of the lesion, the appearance of the intracavitary cyst membrane, and the determination of the density of the lesion^45,50^ (Figure 4). Ultrasonography (US) has limited use in imaging lung HCs. All patients with HCs in the lung on chest x-ray should be evaluated for liver cysts by the abdominal US.^48^ Demonstration of similar cystic lesions in the liver is important both to support the diagnosis and to determine the treatment protocol for this condition. Magnetic resonance imaging (MRI) is not superior to CT in the diagnosis of pulmonary HCs. However, it may be requested in cases with surrounding tissue involvement such as the heart and vertebra. Cyst content can be defined with MRI and the cyst membrane can be monitored. Also, daughter vesicles on MRI may have signal intensity depending on their content. Daughter vesicles are less common in pulmonary cysts than in the liver.^50^

## Bronchoscopy

Bronchoscopy is not routinely used in pulmonary HCs. However, as it may mimic many pulmonary diseases, bronchoscopy can be applied especially in cases where it is necessary for the differential diagnosis of lung cancer. In bronchoscopy, endobronchial bright white-yellow-colored cyst membrane can be observed. If the sample from the cyst fluid is left to stand, a precipitate called hydatid sand, formed by protoscolex and daughter vesicles, may form. A definitive diagnosis is made by demonstrating protoscolex in the cyst fluid. Protoscolices may be demonstrated in cyst aspirate, sputum, or bronchial lavage. In infertile cysts without protoscolex, the laminar membrane of endocysts is strongly stained with PAS.^51,52^

## Diagnosis

Hydatid cyst is suspected in the presence of pulmonary lesions in endemic areas with a history of sheep rearing and exposure to dogs. Radiology and serology are the main diagnostic methods used to confirm the diagnosis. Simple cysts are usually easily diagnosed in endemic areas. However, when cysts rupture and become complicated, they can imitate many diseases, especially lung cancer.^25,26^ In a study performed with pulmonary HC in our clinic, the results of 93 consecutive patients who underwent preoperative IgG ELISA test were evaluated. While the IgG ELISA test was positive in 90.6% of the cases with ruptured cysts, the test was positive in only 12.5% of the cases with intact cysts.^53^

## Differential Diagnosis

When a cystic lesion is detected in a patient from an endemic region, an HC comes to mind first. The most important disease that can be confused with an HC is lung cancer. It can be difficult to differentiate clinically and radiologically in complicated cysts. Other lesions that should be kept in mind in the differential diagnosis include pulmonary abscess, bronchogenic cyst, primary sarcoma of the lung, pulmonary metastases, hematoma, mesothelioma, and granuloma.^50,54,55^

## Management Approach

The treatment of pulmonary HCs is both medical and surgical. Although some studies are reporting the benefit of pharmacotherapy in selected patients, surgical intervention is the main treatment of choice. Medical treatment of pulmonary HC includes benzimidazole group drugs such as albendazole and mebendazole.^45,56^ Very rarely, the membrane of the HC opened into the bronchus can be removed by expectoration and spontaneous healing can be seen.^57^

## Surgery

The main treatment of lung HCs is surgery. The purpose of surgical treatment is to completely remove the parasite, prevent the spread that may occur due to intraoperative cyst rupture, preserve the parenchyma as much as possible, and obliterate the residual space. However, the surgical approach is determined by many factors such as the size of the cyst, whether it is intact or complicated and whether it is multiple or solitary, bilateral or unilateral, and the destruction of the lung parenchyma.^58^

**Figure 5. a,b.**
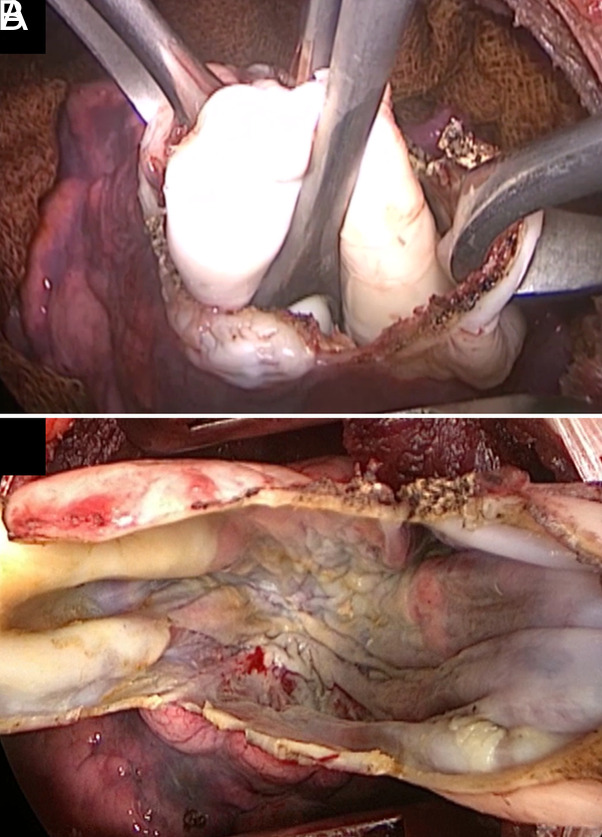
In a case of a giant hydatid cyst, intraoperative cyst membrane removal (a) and cyst cavity after membrane removal (b) are seen.

Various surgical approaches like resection with enucleation, removal of intact cyst after needle aspiration, pericystectomy, wedge resection, segmentectomy, and lobectomy have been reported in the treatment of pulmonary HCs^59^ (Figure 5).

*Enucleation *is the process of removing the cyst with an intact germinative membrane and is suitable for small cysts with a low risk of rupture. A hole is created on the cyst’s adventitia layer to see the underlying white laminated membrane. Then, the anesthesiologist is asked to apply positive pressure ventilation. Positive pressure ventilation helps enucleate cysts.^60^

*Pericystectomy* is the removal of the HC along with the pericyst. Pericyst excision increases the risk of airway leakage and tension pneumothorax in the early period or bronchopleural fistula formation in the late period.^60^

*Barrett's method, *cystotomy with capitonnage, was proffered in 1952. Hydatid fluid is aspirated and the membrane is removed. The cavity is closed by bringing the walls of the cyst closer together using non-absorbable purse-string sutures. Last, the intact parenchymal ends are approximated using non-absorbable sutures. All common approaches are based on the Barrett technique.^61,62^

*The Posadas *method is similar in procedure to the Barrett method, but in that method, the open bronchial airways are closed before capitonnage.^63^

*Cystotomy *is a surgical technique in which only the bronchial openings are closed and the capitonnage procedure is not applied.^59,64^

*Pulmonary resection *is rarely performed in cases with large and unresponsive infected cysts, multiple cysts in a single lobe, bronchiectasis, pulmonary fibrosis, or severe bleeding.^65^

Considering the location of the lesion and ease of access, interventions ranging from thoracotomy incisions of varying sizes to even video-assisted thoracoscopic surgery (VATS) or hybrid interventions are performed, although not as much as open surgery. The use of a double-lumen endotracheal tube is important to prevent aspiration of the cyst contents into the tracheobronchial tree during the operation.^66,67^

### Capitonnage and Uncapitonnage

The most important debate in the surgery of pulmonary cysts is whether obliteration (capitonnage) is required following cystotomy.^68-71^ The capitonnage aims to eliminate the remaining space to prevent long-term air leakage and abscess formation after the surgery. It involves applying a series of purse-string sutures to the cyst wall, starting at its base, until the cavity is completely closed. In most studies, capitonnage is generally advocated for the obliteration of residual space.^68,69^ However, especially in the last 2 decades, studies have been published that argue that capitonnage is not necessary and that only the closure of bronchial leaks will provide healing of the expandable parenchyma.^70,71^ Although there are many studies in the literature on the surgical treatment of pulmonary HCs, comparative studies between the 2 procedures are few and there are no prospective randomized controlled studies published so far.

The capitonnage process is used to obliterate the residual space together with the pericyst and to prevent the formation of postoperative air leakage and empyema.^72^ The authors defending the uncapitonnage technique, on the other hand, state the disadvantage of distorting the pulmonary parenchyma, especially after the removal of multiple cysts and closure of the openings of the major bronchi. It is reported that this situation will result in atelectasis by restricting the re-expansion of the lung after surgery.^59,70,71^ However, there are also studies stating that atelectasis is not seen or seen at a very low rate after the capitonnage process.^73,74^ In addition, another important advantage is that it reduces hospital costs by shortening the hospitalization period in the group without capitonnage.^70^ However, there are also data reporting that the postoperative hospital stay is prolonged and the complication rates are significantly higher in cases without capitonnage.^75,76^ The capitonnage process provides added strength to the lung parenchyma and reduces post-operative air leakage and empyema formation. Therefore, we, like many authors, think that capitonnage should be applied in pulmonary cysts.

### Bilateral Hydatid Cysts

Approximately 15% of pulmonary HCs are bilaterally located.^2,77^ In cases with bilateral cysts, surgical planning should be done by evaluating the cysts' integrity or rupture, their diameters, and the risks of spreading. If there is an intact HC in 1 lung and a ruptured complicated HC in the other lung, surgery should be given priority to the intact cyst. In cases where both lungs are involved, surgical options include simultaneous bilateral thoracotomy/VATS, bilateral staged thoracotomy/VATS, median sternotomy, and Clamshell incision.^77-79^ However, the most appropriate method seems to be sequential thoracotomy or VATS, which provides optimal exposure of the hemithorax. There is usually a 2- to 4-week period between 2 surgeries. A disadvantage of this approach is the potential for a delay between 2 thoracotomies due to slow healing or postoperative complications. The delay may allow the disease to progress, making the final resection more difficult. Longer operation and anesthesia time and postoperative course may be a disadvantage in synchronous thoracotomy. Any surgical procedure has a risk of complications for both lungs. The possibility of complications arising simultaneously in both lungs can be life-threatening. For this reason, it is generally spared to perform bilateral thoracotomy in a single stage.^77-79^

Treatment of bilateral lung cysts with median sternotomy in a single session provides a shorter hospital stay and high postoperative patient comfort. However, it is difficult to reach the cysts, especially in the left lower lobe, and multiple cysts with sternotomy. Complete removal of the cysts is the basic rule in HCs surgery.^78,79^

### Giant Hydatid Cysts

The treatment in giant HCs is the same as in other HCs. However, postoperative complications are more common in giant HCs of the lung since most of them are complicated and are associated with more bronchi than small cysts. Some patients with giant HCs may experience pleural complications and therefore require more comprehensive surgical procedures like decortication.^24,80,81^

Capitonnage is advantageous in cases of giant cysts as it can close multiple airway openings. Nevertheless, the number of bronchial openings and varying degrees of pericystic inflammation may complicate closure with conventional capitonnage sutures. The shrinkage and deformity of the parenchyma after capitonnage of these cysts can be a real problem, especially when dealing with the cavities of giant cysts. In case of parenchymal destruction, the sutures to be applied only in the pericyst will carry the risk of separation and deterioration.^24,82^ For this reason, we prevent the opening of the capitonnage due to inflammation and the development of empyema by entering the pericyst wall and exiting the pleura, with the modified capitonnage method we defined, unlike the traditional purse-string capitonnage.^83^

### Hydatid Cysts in the Liver Dome

It is reported that the liver and the right lung are involved together in 4%-25% of cases with HCs.^84^ In these cases, a single-session transthoracic approach saves the patient from a second surgery or invasive intervention. These cases are approached by thoracotomy from the sixth, seventh, or eighth intercostal space, depending on the cyst's location in the lung. First of all, surgical intervention is performed for the cyst in the lung. A radial incision is made to the diaphragm to reach the liver cyst. The cyst membrane is removed by aspirating the cyst fluid. Bile leakage is controlled and the leaking areas are sutured. If a giant cyst is present, it may be considered to fill the cavity with the omentum. In smaller cavities, the cavity can be reduced by approximating the cyst wall with sutures. Before the diaphragm is closed, a pezzer drain is placed in the cavity and the drain is taken out from the abdominal wall. The opening in the diaphragm is closed using nonabsorbable sutures.^84^

In selected cases with liver, spleen, or multiple intra-abdominal cysts requiring exploratory laparotomy, an intra-abdominal approach to lung cysts may be an option. This surgical approach, in which the above procedure is performed in reverse, is rarely required, and if indicated, it must be performed by an experienced thoracic surgeon.^84,85^

### Pulmonary Resection for Hydatid Cyst

Preservation of the lung parenchyma is one of the basic principles of HC surgery. Lung resections are rarely needed for the treatment of pulmonary HCs. However, cyst rupture may lead to infection, resulting in abscess formation and parenchymal destruction. In this case, lung resection in the form of a wide wedge resection or even a lobectomy may be required. Another indication for anatomical resection is the presence of a complicated HC indistinguishable from lung cancer or aspergilloma.^86,87^ In the literature, lobectomy is recommended in cases where a cyst contains more than 50% of the lobe, infected cysts that do not respond to treatment, multiple cysts in a single lobe, and bronchiectasis, pulmonary fibrosis, or severe bleeding.^19,86,87^

In the literature, the rate of lobectomy in giant cysts ranges from 0.5% to 54.5%.^19,63,74,86^ Onal and Demir^19^ reported that they treated all 32 children with giant HCs with the cystotomy–capitonnage method and they did not apply pulmonary resection to any of the cases. Given the high healing capacity of the lung tissue, the atelectasis, existing infection, and parenchymal damage should be given a chance to heal. We believe that lung resection should not be considered as much as possible, regardless of the size of the cyst, even in complicated cysts, especially in children and young adults.

### Outcomes After the Surgical Approach

Generally, surgery in pulmonary HCs has satisfactory results. However, many postoperative complications can be seen, most of which do not cause serious problems. Postoperative complications are affected by the type of operation and the number and size of cysts. Postoperative complications are expected more in giant cysts and complicated cysts. Various studies have reported a postoperative complication rate of up to 20% and a mortality rate of 0%-2% in patients with pulmonary HCs.^19,23,24,82,87^

## Antiparasitic Therapy

Indications for medical treatment include patients for whom surgery is contraindicated, smaller cysts, patients who refuse surgery, multiple organ involvement, recurrent cysts, multiple cysts, and patients with intraoperative HCs fluid spillage.^45^

Mebendazole and albendazole have been used in the treatment of HCs since the 1980s. In many studies, albendazole is more effective than mebendazole.^9,88^ Albendazole is used for a shorter time than mebendazole but still has a better response. Its favorable pharmacokinetic profile allows it to reach higher concentrations in serum and cyst fluid. In addition, albendazole sulfoxide, the hepatic metabolite of albendazole, is also an active agent against the parasite. Although the method of use is 10-15 mg/kg/day divided into 2 doses per day, there is no definite standard dose and treatment period.^88,89^ The efficacy of praziquantel in clinical trials has been variable and has not yet been demonstrated to have a definitive role in primary drug therapy. Praziquantel has been used alone or in combination with albendazole.^90^

The most general side effects associated with the use of albendazole are headache and increased liver enzymes in 10%-20% of patients. Other serious side effects include leukopenia, anemia, thrombocytopenia, and pancytopenia and patients with pre-existing liver damage or dysfunction are at higher risk of developing these conditions. Some other side effects of albendazole include abdominal pain, vomiting, fever, and nausea. A hypersensitivity reaction to the drug like urticaria and pruritus may occur in a minority of patients.^88,89^

Usluer et al^91^ studied the effect of preoperative administration of albendazole on the cuticular membrane in lung HCs. They showed that the tensile strength values of the cuticular membrane of the cysts excised were statistically significantly lower in cases who received preoperative 3 cycles of peroral 10 mg/kg/day albendazole treatment compared to those who did not.

Aggarwal and Wali^56^ reported that only 1 of the 10 patients they treated with albendazole had mild symptomatic improvement, while the symptoms continued in the other 9 cases, and no radiological improvement was seen in any of the cases. Keramidas et al^92^ reported complications in 11 cases after albendazole treatment in their 36 pediatric pulmonary cyst study. Most complications occurred approximately 2 months after the start of treatment, and all affected patients required surgical treatment. In addition, it has been reported that surgery becomes more difficult and complications increase in these cases. Albendazole treatment is highly effective in preventing relapses and the recurrence rate was significantly higher in patients who do not albendazole.

Preoperative albendazole therapy weakens the walls of pulmonary cysts and can cause their rupture, leading to complications. For this reason, it should not be administered to patients who are candidates for surgical treatment. We prefer to use albendazole treatment routinely in our clinic to prevent recurrences in the postoperative period. Recurrence was observed in only 1 of 153 patients who received albendazole treatment at a dose of 15 mg/kg/day in 2 postoperative 15-day cycles.^89^

## Conclusion

Pulmonary HCs are more prevalent in children than adults. Surgical treatment should be applied as soon as possible after diagnosis. Patients with giant and complicated cysts usually have a complex clinical picture and these lesions carry a greater risk of postoperative complications than small cysts. In surgery, lung parenchyma should be preserved as much as possible. Medical treatment should be used in the postoperative period to prevent recurrences.
